# Children’s Body Odors: Hints to the Development Status

**DOI:** 10.3389/fpsyg.2020.00320

**Published:** 2020-03-04

**Authors:** Laura Schäfer, Agnieszka Sorokowska, Kerstin Weidner, Ilona Croy

**Affiliations:** ^1^Department of Psychotherapy and Psychosomatic Medicine, Technische Universität Dresden, Dresden, Germany; ^2^Institute of Psychology, University of Wrocław, Wrocław, Poland

**Keywords:** olfaction, bonding, puberty, chemosignal, body odors, parent–child relationship, age

## Abstract

Mothers can recognize their own children by body odor. Besides signaling familiarity, children’s body odors may provide other information relevant to maternal caregiving behavior, such as the child’s developmental status. Thus, we explored whether mothers are able to classify body odors on pre- vs. postpubertal status above chance levels. In total, 164 mothers were presented with body odor samples of their own and four unfamiliar, sex-matched children who varied in age (range 0–18 years). Pubertal status was measured by (a) determining the child’s steroid hormone level and (b) parental assessment of the child’s developmental stage using the Pubertal Development Scale. Mothers classified developmental status with an accuracy of about 64%. Maternal assessments were biased toward pre-puberty. Classification was predicted by perceptual evaluation of the body odor (i.e. intensity and pleasantness) and by the child’s developmental stage, but not by hormones. In specific, mothers with pubertal-aged children classified body odors using the child’s developmental status, whereas mothers with younger children only classified body odors using perceptual information (i.e. intensity and pleasantness). Our data suggests that body odors convey developmental cues, but how this developmental information is manifested in body odor remains unclear.

## Introduction

Body odors are a potent chemosignal in human social communication for two reasons. First, they allow recognition of the own relative among a number of individuals (Pause et al., 1998; Lundström et al., 2009). Second, both hedonic [i.e. pleasantness or attractiveness, (Kuukasjärvi et al., 2004; Croy et al., 2017)] ratings and neural activity (Cecchetto et al., 2019) support the idea that body odors communicate affective information to recipients. Both of these features of body odors are highly relevant in the context of mother–child bonding. In specific, kin recognition serves to facilitate a targeted investment of resources (Burnstein et al., 1994; Chapais et al., 2001), which is important for providing one’s offspring with care. With regard to the affective value, in a previous study asking for parental perception of their children’s body odors, we found that a baby’s body odor was perceived as highly adorable and pleasant (Croy et al., 2017). In addition, mothers respond to infant’s body odors with neural activation in reward-related processing areas [e.g. neostriate areas (Lundström et al., 2013)]. The authors concluded that the infantile odor may evoke a desire to bond in parents.

Kin recognition has been demonstrated in response to infants, preschool, and adolescent children (Porter et al., 1983; Weisfeld et al., 2003; Ferdenzi et al., 2010). Besides, recognition and a mother’s preference for the body odor of her own child seem to affect each other. For example, mothers who are not able to recognize their own child’s body odor do not show a preference for their child’s odor. Consistent with this, Croy et al. (2019) showed that mothers with postpartum bonding disorders had a lower preference for their own child’s body odor, compared to healthy controls. Further, in a recent study conducted in our lab, we presented 164 healthy mothers to body odor probes of their own and sex-matched unfamiliar children in different age groups, from infancy to adulthood (Schäfer et al., in press). Interestingly, the relationship between source of the body odor (i.e. child vs. other) and odor preference in mothers, varied across the child’s development – i.e. mothers preferred their own child’s odor when the child was pre- or postpubertal, but not when the child was in early puberty. In that stage, the decrement in maternal pleasantness ratings of their son’s body odor was associated with increasing testosterone levels in their sons. In addition, mothers were not able to identify their own child’s body odor around puberty but were able to do so in pre- and late pubertal stages. Such findings, led to two suppositions; (1) that the loss of kin recognition with initial hormonal release around puberty is causal for a mother’s lack of preference to her child’s body odor and (2) that kin recognition and preference of the odor recover over time, because mothers get used to (i.e. are able to identify) the odor again.

In general, developmental cues are necessary for signaling a certain stage of maturity, which affects the amount and the manner of caregiving exerted by parents on their children. Several infantile facial characteristics facilitate a perception of cuteness, and thus elicit approach and attachment behavior (Kringelbach et al., 2016). Those features are lost with increasing development status and in the same time willingness for parental investment declines (Volk et al., 2007). In the domain of olfaction, similar mechanisms may be present.

In order to serve as a developmental cue, it is a prerequisite that body odors change during development. These changes are presumably due to developmental hormones. We base this assumption on the observation that female body odors smell different across the menstrual cycle. In specific, men rate female body odors as more pleasant during ovulation (Havlíček et al., 2017), and this preference is disturbed by women’s hormonal contraceptive use (Kuukasjärvi et al., 2004). The particular hormones that mediate this alteration in odor preference across the menstrual cycle are yet to be identified but steroid hormones may be a likely candidate. Steroid hormones seem to affect body odor perception – for example, higher estradiol concentration is associated with higher attractiveness of female body odor (Lobmaier et al., 2018), whereas male body odor contains more androgen-derived steroids and is perceived as more intense (Sergeant, 2010). The relation to actual testosterone levels has however been unclear (Rantala et al., 2006).

As short-term hormonal fluctuations, such as those present during the menstrual cycle, are perceivable via body odor, we also assume that slow, long-term changes of hormonal and pubertal development from infancy (prepubertal stage) to adulthood (postpubertal stage) is reflected in body odor perception. Support for this supposition comes from a questionnaire study asking for parent’s evaluation of their children’s body odors across development (Croy et al., 2017). Parents reported less pleasantness of odors from their pubertal compared to younger children, which might mirror the increase of steroid hormones during that period.

Puberty is characterized by two main stages of development – the first stage, adrenarche, occurs between the age of 5 and 9 years and is characterized by arise of androgens without leading to visible changes. Children in that phase are still referred to as prepubertal. The second stage, gonadarche, begins between 9 and 11 years and is marked by testosterone and estradiol increase. During that phase, primary and secondary sexual features develop, peaking with transition to adulthood (Dorn et al., 2006).

The present study aimed to address whether body odors function as an indicator for development and explored the ability of mothers to identify a child’s developmental stage, using body odor. We hypothesized that mothers are able to accurately distinguish pre- from postpubertal odors (H1). Further, we assumed that this ability depends on developmental familiarity of the mothers: a mother of a prepubertal child might be particularly good at accurately detecting prepubertal status in body odor, whereas a mother of a postpubertal child might be better able to classify postpubertal body odors (H2). Finally, we explored potential mechanisms (maternal perceptual ratings, hormonal and developmental status of the child) contributing to developmental classification of body odor (H3).

## Materials and Methods

The study was approved by the Ethics Committee of the University of Dresden (Code: EK 104032015), and all participants provided written, informed consent in accordance with the Declaration of Helsinki. The study was part of a broader project assessing maternal kin recognition and hedonic evaluation of children’s body odors (including the dimensions sweetness, wanting, and attraction) in relation to genetic analysis of the human leukocyte antigen complex. In order to facilitate readability, we omit from presenting the whole study here and focus on presentation of parts relevant for the current research question. For all further information, please compare (Schäfer et al., in press).

### Participants

The sample consisted of *N* = 164 mothers (*M* = 37.5, *SD* = 7.8) with *N* = 226 children (*M* = 7.6, *SD* = 5.9 years, *n* = 124 girls, *n* = 102 boys), of whom 226 BO probes were sampled. Inclusion criteria was being the biological mother of a child between 0 and 18 years of age. Current pregnancy, insufficient knowledge of German language and anosmia or hyposmia were exclusion criteria. Olfactory performance was assessed prior to study inclusion with a short version of the standardized Sniffin Stick’s Step II^®^ screening for olfactory identification ability (Lötsch et al., 2016). In addition, prior to the experiment mothers were asked if they had acute rhino-sinonasal disorders (which could impair olfactory abilities), and were postponed to a later date if they reported having so.

### Study Procedure

Participants came to an initial meeting in the lab of the Department of Psychosomatics at the University Hospital Dresden, in which the study procedure was explained and inclusion and exclusion criteria were tested. After meeting those criteria, participants were equipped with a study kit for sampling the body odors and hormonal status of their children at home.

The study kit included odorless shower gel, odorless detergent, a salivette (Salivette^®^, code blue, SARSTEDT AG & Co. KG, Nümbrecht, Germany), an unworn 100% cotton t-shirt or onesie in the respective size of the child, a re-closeable plastic zip bag, and a study protocol. In order to minimize potential sources of smell, the garment had been washed by the experimenter with an odorless detergent. The protocol contained detailed instructions for body odor and hormonal sampling, and also screened for potential confounders of the body odor sample – i.e. the presence of contamination of the sample (e.g. urine or feces), the medical condition of the child (use of drug and current illness), and the situation at home (smoking, pets, and number of persons who sleep in the children’s room).

#### BO Sampling

The children slept for one night in the garment. Prior to that, parents were instructed to wash sheets and clothes additionally worn to the garment with odorless detergent (Denkmit Vollwaschmittel Ultra Sensitive, dm-drogerie markt GmbH & Co. KG, Karlsruhe, Germany^1^) and the children were asked to shower with the odorless shower gel (both EUBOS flüssig wasch+dusch, Dr. Hobein GmbH, Meckenheim, Germany^2^), as well as to refrain from usage of any perfumed hygiene products. After wearing the garment for one night, the sample was stored in a re-closeable plastic zip bag and brought back to the lab by the parents the next morning, which was where the sample was cut in half and then frozen (−25°C) until the experiment was carried out.

#### Hormonal Sampling and Assessment of Development Status

For all children aged between 5 and 18 years, hormonal sampling and maternal assessment of the pubertal status using the Pubertal Development Scale [PDS, (Watzlawik, 2009)] was performed. Hormonal sampling was carried out in the evening before the experimental night in order to measure hormonal status in direct relation to the body odor sample. Mothers were instructed to explain their children to chew for 60 s on the salivette until it contained sufficient saliva. Overnight, the salivette was stored in the fridge and the next morning, saliva and body odor sample were taken to the lab where they were frozen at −25°C until analyses. Hormonal analysis was carried out by the Dresden LabService GmbH. For each sample, testosterone and estradiol concentration was determined via immune-assay analyses as follows (Rohleder et al., 2006).

Concentration of alpha-amylase in saliva was measured by an enzyme kinetic method: saliva was processed on a Genesis RSP8/150 liquid handling system (Tecan, Crailsheim, Germany). First, saliva was diluted 1:625 with double-distilled water by the liquid handling system. Twenty microliters of diluted saliva and standard were then transferred into standard transparent 96-well microplates (Roth, Karlsruhe, Germany). Standard was prepared from “Calibrator f.a.s.” solution (Roche Diagnostics, Mannheim, Germany) with concentrations of 326, 163, 81.5, 40.75, 20.38, 10.19, and 5.01 U/l alpha-amylase, respectively, and bidest water as zero standard. After that, 80 ml of substrate reagent (α-amylase EPS Sys; Roche Diagnostics, Mannheim, Germany) were pipetted into each well using a multichannel pipette. The microplate containing sample and substrate was then warmed to 37°C by incubation in a water bath for 90 s. Immediately afterward, a first interference measurement was obtained at a wavelength of 405 nm using a standard ELISA reader (Anthos Labtech HT2, Anthos, Krefeld, Germany). The plate was then incubated for another 5 min at 37°C in the water bath, before a second measurement at 405 nm was taken. Increases in absorbance were calculated for unknowns and standards. Increases of absorbance of diluted samples were transformed to alpha-amylase concentrations using a linear regression calculated for each microplate (GraphPad Prism 4.0c for MacOSX, GraphPad Software, San Diego, CA). The intra- and interassay coefficients for amylase were below 9 and 9%,respectively. The detection threshold for the analyzed samples was at 0.3 pg/ml for estradiol and at 1.8 pg/ml for testosterone.

Mothers completed the PDS (Watzlawik, 2009) which is a standardized assessment of pubertal status with sufficient reliability (*r* = 0.64–0.69) and validity (self- vs. external assessment, *r* = 0.39 and 0.83) (Watzlawik, 2009). The PDS comprises three questions for each boys and girls (development of body hair, growth of breast/beard, menarche, and voice break) which are summed up to a score indicating pubertal status (ranging from 3 = puberty has not begun) to 12 (development completed). According to the manual (Crockett and Petersen, 1987; Crockett, 1988; Carskadon and Acebo, 1993), the following categories were defined as indicators for the pubertal status of boys: prepubertal (3 points), early pubertal (4 or 5 points), midpubertal (6, 7, or 8 points), late pubertal (9–11 points), and postpubertal (12 points) status. For girls the classification was: prepubertal (2 and no menarche), early pubertal (3 and no menarche), midpubertal (>3 and no menarche), late pubertal (<7 and menarche), and postpubertal (8 and menarche) status.

#### Experimental Procedure

One and half hours before the experimental session, body odor samples were thawed. Subjects were asked to refrain from eating, drinking coffee, and smoking 1 h prior to the testing, as well as from usage of perfume on the study day. The experimenter refrained from usage of perfume and wore rubber gloves in order to not confound the odor of the samples.

In total, the mothers assessed six body odor samples including the body odor of the own child and four body odor probes of unfamiliar children, as well as an unworn blank probe (previously washed with the odorless detergent) to control for intensity of the body odor samples. The unfamiliar children were matched to the same sex as the own child and two different age groups (two children of the same age group as the own child, two children of a different developmental group; i.e. a prepubertal age group when the own child was postpubertal, and vice versa).

For body odor presentation, the experimenter instructed the subject to close the eyes during 6 s of smelling in order to focus on the smell and to not be biased by seeing if the sample belonged to a t-shirt or to a onesie. The sample was placed by the experimenter directly under the nose of the participants, with the armpit pad upward. After 6 s, the probe was placed back and the subject had to open her eyes and to rate the body odor.

Prior to the rating procedure, body odors were presented in a test trial without assessment of the probes. This was done in order to anchor the probes for intensity. The six samples were then rated on pleasantness and intensity using visual analogue scale (VAS), ranging from 0 (“not at all”) to 100 (“very”). Afterward, mothers rated the age group of the body odor donor. Therefore, the subjects were instructed to choose one of the following categories for each sample: “<1 year,” “1–3 years,” “4–8 years,” “9–13 years,” “14–18 years,” and “>18 years.”

#### Statistical Analyses

All statistical analyses were performed with IBM SPSS Statistics 25 (IBM Corp, 2017).

For analyses, three age categories [based off Dorn et al. (2006)] were created to indicate the child’s developmental status. These were as follow – prepubertal (0–8 years), midpubertal (9–13 years), and postpubertal (≥14 years). This grouping was confirmed by the prior assessed PDS categories. Almost all (126 out of 128, 98.4%) children aged 0–8 years had a PDS score which indicated prepuberty and 50 out of 55 (90.9%) of the children aged 14–18 years had a PDS score which indicated a late or postpubertal stage. We decided to exclude body odor probes of those seven children whose age groups did not align with the PDS for statistical analysis of H1 and H2. We also decided to exclude body odor probes of the *n* = 42 midpubertal children (9–13 years), as this group comprised children of heterogeneous developmental status at the transition between pre- to postpubertal status, and therefore was not suitable to be classified in one consistent stage (see Table 1).

**TABLE 1:** Frequencies of all presented body odor samples classified by PDS category and age group.

	Age group					
	
PDS category	Prepubertal (0–8 years)	*n* (%) girls	Midpubertal (9–13 years)	*n* (%) girls	Postpubertal (14–18 years)	*n* (%) girls
Prepubertal	126 (98.4%)	67 (53.2%)	15 (35.7%)	4 (26.7%)	0	0
Early pubertal	2 (1.6%)	2 (100%)	10 (23.8%)	2 (20.0%)	0	0
Midpubertal	0	0	10 (23.8%)	9 (90.0%)	5 (9.4%)	0 (0.0%)
Postpubertal	0	0	7 (16.7%)	4 (57.1%)	50 (90.9%)	35 (70%)

**N* = 226 children, *n* = 124 girls, *n* = 102 boys, *n* (%) girls = number and percentage of girls within the respective category.*

This procedure led to a final sample size of 177 body odor probes for analysis of H1 and H2. As each mother rated multiple body odor samples, this resulted in 890 maternal assessments of developmental stage. For analyzing H3, we used the total sample of 226 body odor probes (=1127 assessments).

All analyses were carried out (a) for all children and (b) only for unfamiliar children excluding the own child’s body odor sample from analyses. This additional analysis was done in order to not bias performance due to recognition of the own child’s odor and thus assuming to know the age. For reasons of clearness, only analyses for all children are presented here. Results regarding the unfamiliar children are listed in the Supplementary Material (see Supplementary Figures 3–5 and Supplementary Tables 1–3).

##### Mothers are able to accurately distinguish pre- from post-pubertal odors (H1); classification ability depends on developmental familiarity of the mothers (H2)

We first assessed whether there was a significant difference of maternal classification in children of prepubertal vs. postpubertal stage using χ^2^ test. Subsequently, we tested the sensitivity, specificity and accuracy of classification. Therefore, all maternal answers were categorized in one 4-field matrix for each developmental status, and this was based on their accuracy. The four categories are as follow – (1) a true positive (tp) or hit was assigned in case of correct detection of the developmental status, (2) a true negative (tn) was assigned when a mother correctly rejected the developmental status (e.g. not choosing prepubertal for a postpubertal body odor), (3) a false positive (fp) was assigned when a postpubertal sample was rated as prepubertal (or vice versa), and (4) a false negative (fn) was assigned, when a body odor sample was not detected as pre- or postpubertal even though it was pre-/postpubertal. We calculated sensitivity, specificity, and accuracy of the maternal classification for each developmental status. Additionally, we calculated the RATZ-index indicating how much the maternal hit rate increases compared to the chance level [relative increase of the hit rate compared to the random hit rate (Marx and Lenhard, 2010)]. The index can take values between 0 and 1, with values from 0.3 being seen as an improvement to the random rate.

In order to explore the impact of maternal developmental familiarity, we compared for each mother the classification of those body odor samples which had the same developmental status as the own child (developmental familiar classification) to the classification of those body odor samples which had a different developmental status as the own child (developmental unfamiliar classification). Classification performance across the groups was compared using a 4 × 2 χ^2^ test calculator^3^.

We tested the influence of hormonal contraceptive use on maternal classification performance, as this has been previously reported to influence olfactory perception (Derntl et al., 2013). On the day of testing, 38.5% of the mothers stated to use hormonal contraception, 54% stated not to use hormonal contraception, and 7.5% did not reply to this question. Comparison between the groups revealed no significant differences between the groups [χ^2^ (1) = 5.70, *p* = 0.127], which is why we did not include this in further analyses. We also compared maternal classification performance for boys and girls within each developmental status, and found no significant differences [prepubertal classification: χ^2^ (1) = 3.65, *p* = 0.057; postpubertal classification: χ^2^ (1) = 0.10, *p* = 0.757]. Therefore, we did not perform any further sex-specific analyses.

##### Predictors of pre- vs. postpubertal body odor classification (H3)

For H3, logistic regression analyses including bootstrapping (*n* = 1000) were performed with the binary outcome of pre- vs. postpubertal maternal classification as dependent variable.

As predictors we modeled perceptual evaluation of the body odor (pleasantness and intensity) in order to assess the influence of affective perception on the classification. For exploring the influence of developmental cues on body odor classification, the PDS score and hormonal status (comprising the testosterone status for boys and the estradiol status for girls in pg/ml) were included as further predictors. All predictors were tested in one model using enter method.

All analyses were performed across all children and all mothers and then for developmental familiar samples and developmental unfamiliar samples separately.

## Results

### Mothers Are Able to Accurately Distinguish Pre- From Post-pubertal Odors (H1)

**FIGURE 1:**
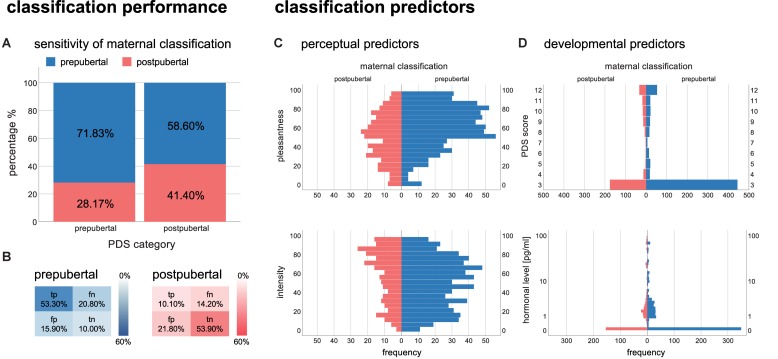
Left panel: classification performance: **(A)** percentage of the sensitivity of maternal classification plotted by PDS categories; **(B)** percentage of frequency of true positives (tp), false positives (fp), false negatives (fn), and true negatives (tn) plotted in blue for prepubertal and in read for postpubertal body odors. Color intensity indicates frequency of choice. Right panel: classification predictors: **(C)** perceptual predictors (above: pleasantness, below: intensity); **(D)** developmental predictors [above: pubertal development scale (PDS), below: hormonal concentration in pg/ml, estradiol for girls, testosterone for boys]. Assessment of developmental predictors was carried out for all children from the age of 5 years on and therefore children under the age of 5 exhibit a value of 3 for the PDS (prepubertal) and a value of 0 for the hormonal concentration.

When presented to body odors of prepubertal children, mothers stated in 71.6% of the cases that those odors were from a prepubertal donor and in 28.17% that these odors were from a postpubertal donor. When presented to body odors of postpubertal children, mothers stated in 58.6% of the cases that those odors were from a prepubertal donor and in turn, mothers stated in 41.4% of the cases that the odors were from a postpubertal donor (see Figure 1). The classification of an odor as postpubertal was significantly higher when mothers were presented to postpubertal odors than when they were presented to prepubertal odors [χ^2^ (1) = 10.82, *p* = 0.001]. Furthermore, this result reveals that BOs are more frequently rated as originating from a prepubertal than from a postpubertal donor.

The detection of prepubertal odors was performed with an accuracy of 63%. This value exceeds the 50% chance level. However, the RATZ-index of 0.11 is rather low and suggests that mothers do not perform substantially better than chance. Maternal assessments of prepubertal odors had a sensitivity of 72.0% and a specificity of only 38.7%, indicating that maternal assessments tended to accept the classification of a sample as prepubertal [χ^2^ (1) = 472.63, *p* < 0.001].

A similar effect was found for postpubertal body odors, which were detected with an accuracy of 64.0% at an RATZ-index of 0.14. Maternal assessments of postpubertal odors had a sensitivity of only 41.4% and a specificity of 71.2%, indicating that maternal assessments tended to reject the classification of a sample as postpubertal.

### Classification Ability Depends on Developmental Familiarity of the Mothers (H2)

Separate analyses of developmental familiar samples and developmental unfamiliar samples revealed that mothers were more accurate in classifying body odors of donors at the same developmental status as their own child (see Supplementary Figures 1, 2).

Hence, mothers of prepubertal children could identify prepubertal odors with a higher accuracy of 65.2% (RATZ-index = 0.19; sensitivity = 74.4%; specificity = 43.8%) compared to the 60.6% accuracy of mothers having postpubertal children (RATZ-index: 0.04%; sensitivity = 67.7%; specificity = 35.7%). The difference between maternal classification of developmental familiar samples and developmental unfamiliar samples was significant [χ^2^ (1) = 9.84, *p* = 0.020].

Similarly, mothers of postpubertal children were more accurate in classification of postpubertal body odors (developmental familiar samples: accuracy = 65.2%; RATZ-index: 0.19; sensitivity = 43.2%; specificity = 73.6%; developmental unfamiliar samples: accuracy = 62.2%; RATZ-index: 0.07%; sensitivity = 37.7%; specificity = 68.1%) and maternal classification differed significantly between both groups [developmental familiar samples vs. developmental unfamiliar samples: χ^2^ (1) = 8.95, *p* = 0.029].

### Predictors of Pre- vs. Postpubertal BO Classification (H3)

The overall regression model across all mothers was significant [χ*^2^* (4) = 79.98, *p* < 0.001], revealing that pleasantness (*p* < 0.001), intensity (*p* < 0.001), and pubertal status (PDS score, *p* = 0.007) predicted developmental classification, while hormones did not relate to maternal decision (*p* = 0.952, see Table 2). In particular, higher pleasantness predicted prepubertal classification, whereas higher intensity and higher pubertal status were associated with postpubertal classification (see Figure 1).

**TABLE 2:** Results of logistic regression model predicting age classification; β, SE, Wald, df, *p*, *e*^β^, 95% CI (*e*^β^) of each predictor: all samples.

Predictor	β	SE β	Wald’s χ^2^	df	*p*	*e*^β^	95% CI (*e*^β^)	
Pleasantness	−0.018	0.003	34.685	1	0.000	0.982	0.976	0.988
Intensity	0.013	0.003	23.358	1	0.000	1.014	1.008	1.019
Pds	0.062	0.023	7.212	1	0.007	1.064	1.017	1.114
Hormones	0.000	0.005	0.004	1	0.952	1.000	0.989	1.01
Constant	−0.848	0.284	8.928	1	0.003	0.428		

**R*^2^ = 0.08 (Cox and Snell) and 0.12 (Nagelkerke). Model χ^2^ (4) = 79.98, *p* < 0.001.*

The further regression models testing the respective groups were significant for developmental familiar samples [χ^2^ (4) = 38.62, *p* < 0.001] and for developmental unfamiliar samples [χ^2^ (4) = 50.29, *p* < 0.001]. For classification of developmental familiar samples, pleasantness, (*p* = 0.001), intensity (*p* = 0.001), and pubertal status (*p* = 0.001) but not hormonal status (*p* = 0.706) predicted developmental classification (see Table 3). Higher pleasantness related to prepubertal classification, whereas higher intensity and higher pubertal status were associated with postpubertal classification. For classification of developmental unfamiliar samples, only the perceptual ratings, pleasantness (*p* < 0.001) and intensity (*p* = 0.001), emerged as significant predictors with higher pleasantness predicting pre-, and higher intensity predicting postpubertal classification (see Table 4 and Supplementary Figures 1, 2).

**TABLE 3:** Results of logistic regression model predicting age classification; β, SE, Wald, df, *p*, *e*^β^, 95% CI (*e*^β^) of each predictor: developmental familiar samples.

Predictors	β	SE β	Wald’s χ^2^	df	*p*	Exp (*B*)	95% CI (*e*^β^)	
Pleasantness	−0.013	0.004	11.251	1	0.001	0.987	0.979	0.994
Intensity	0.012	0.004	11.426	1	0.001	1.013	1.01	1.02
Pds	0.092	0.028	10.519	1	0.001	1.096	1.04	1.16
Hormones	0.003	0.007	0.142	1	0.706	1.003	0.990	1.02
Constant	−1.240	0.373	11.075	1	0.001	0.289		

**R*^2^ = 0.07 (Cox and Snell) and 0.10 (Nagelkerke). Model χ^2^ (4) = 38.62, *p* < 0.001.*

**TABLE 4:** Results of logistic regression model predicting age classification; β, SE, Wald, df, *p*, *e*^β^, 95% CI (*e*^β^) of each predictor: developmental unfamiliar samples.

Predictors	β	SE β	Wald’s χ^2^	df	*p*	Exp (*B*)	95% CI (*e*^β^)	
Pleasantness	−0.026	0.005	26.491	1	0.000	0.974	0.964	0.994
Intensity	0.014	0.004	10.826	1	0.001	1.014	1.006	1.023
Pds	0.001	0.042	0.000	1	0.990	1.001	0.922	1.086
Hormones	−0.004	0.010	0.182	1	0.670	0.996	0.977	1.015
Constant	−0.160	0.454	0.124	1	0.725	0.852		

**R*^2^ = 0.12 (Cox and Snell) and 0.17 (Nagelkerke). Model χ^2^ (4) = 50.29, *p* < 0.001.*

## Discussion

The present findings highlight that maternal classification of the body odor changes depending on the pubertal stage of the child. Further, accuracy of maternal classification was moderately low (i.e. around 64%). In detail, we observed a high sensitivity and low specificity in detection of prepubertal status and vice versa – i.e. postpubertal classification corresponded to low sensitivity and a high specificity. Hence, mothers were more prone to identify the presented body odors as prepubertal rather than postpubertal.

Mothers performed better when assessing developmental familiar samples than when assessing developmentally unfamiliar samples. This finding may indicate that mothers being exposed to a certain developmental stage are able to incorporate developmental knowledge better. This is illustrated by analysis of the classification’s determinants – i.e. perceptual evaluation of the body odor, as well as the assessed pubertal status predicted the maternal choice. In particular, the developmental familiar classification was guided by perceptual ratings and developmental information, whereas mothers based their decision on perceptual assessment only when rating developmentally unfamiliar samples.

The overall accuracy of developmental classification was low, although exceeding chance level. Body odors consist of various components including rather stable factors, such as the genetic profile (Milinski et al., 2013), but also highly variable influences, such as food, culture (Havlíček et al., 2017), or disease (Olsson et al., 2014). It is unclear how much variance each of these factors explain in odor perception. Typically, odors are difficult to identify in an unaided identification task and susceptible to label effects (Cuevas et al., 2009; Herz, 2003), which explains why odor perception is often ambiguous. Considering those facts, the low odor-identification accuracy found in this study is not surprising. Nonetheless, our data suggest that body odors at least carry the potential to signal developmental stage, which is explained in the following paragraphs.

The maternal susceptibility of detecting prepubertal status suggests that body odors serve as an important signal in human chemical communication. This appears especially true in infancy, when children are dependent on parental care. Parenting in the early childhood is characterized by formation of attachment, enabling the child to survive safely and to develop healthily in the world (Bowlby, 1958). Infantile positive signals, such as a cute baby face or babbling, trigger brain correlates of reward and approach behavior (Kringelbach et al., 2016). This is assumed to apply for body odors as well, and indeed, a baby’s body odor elicits reward on a neural level, especially to mothers (Lundström et al., 2013). In our data, prepubertal status was detectable above chance by all mothers, independent from their expert status, which suggests that an infantile body odor may also serve as a universal cue for cuteness, similar to the “Kindchenschema.” If this effect were to exist, it might have contributed to the maternal tendency to classify a body odor as prepubertal (rather than postpubertal), observed in this study. Further from an evolutionary perspective, our results may reflect a primacy to interpret children’s body odors first as a general “cuteness.” We assume that body odor perception leads to neural and behavioral responses similar to those observed for the “Kindchenschema” – i.e. a set of responses targeted to ensure the child’s survival by formatting a bond that is prioritized over detachment (Glocker et al., 2009). Preliminary fMRI data from our lab indeed indicate that babies’ body odors elicit neural correlates in the maternal brain similar to those reported for facial cuteness (Schäfer et al., 2019). However, further studies investigating the perception of infantile body odors across parents (including fathers) and non-parents still need to clarify the universality of such a stimulus.

Besides cuteness, odors may also communicate a certain degree of maturity. While maternal sensitivity for detecting postpubertal status was lower than for prepubertal status, postpubertal recognition was characterized by a higher specificity. These findings suggest that body odors change with increasing development, – however, which particular features determine this change and drive olfactory perception remains unclear. We did not observe any influence of steroid hormones on age classification. We know from our previous data that steroid hormones can affect maternal evaluation of pleasantness, however this finding is only apparent for male children in the transition from pre-to post-pubertal status [9–13 years (Schäfer et al., in press)].

We did not observe sex-related differences in maternal classification for postpubertal children. However, an important limitation is that we did not assess the menstrual cycle phase of postpubertal girls, which is known to affect body odor assessment (Havlíček et al., 2017). This should be regarded in further studies.

Salivary steroid hormones were measured in this study. These hormones fluctuate across the day (Landman et al., 1976) and do not always relate to secondary sexual features (Shirtcliff et al., 2009). Nevertheless, it is assumed that steroid hormones indicate maturity in the transition phase when the external development is not yet complete (Dorn et al., 2006). Based on our study we cannot exclude that steroid hormones are perceivable in body odor, further studies using different sampling methods may lead to different effects. Here, the external manifestation of pubertal development affected body odor classification, as children of higher pubertal status were more often classified as postpubertal. Further, this effect was driven by the mothers having experience with postpubertal children. As the onset of puberty is complex and characterized by various endocrinological cascades (Grumbach, 2002), we do not know if hormones other than steroids change body odor composition and further promote postpubertal recognition. The need of chemosensory body odor profiling is hence obvious in order to determine volatile odorants, which constitute body odor and affect hedonic evaluation.

As our study points out, perceptual assessment was a strong predictor for age classification across all mothers. Pleasantness was related to prepubertal classification, which is in line with previous findings on positive evaluation of infant’s body odor (Fleming et al., 1993; Okamoto et al., 2016; Croy et al., 2017, 2019). Moreover, pleasantness perception of an infant’s odor is an important cue mediating parental care as it facilitates affectionate love (Okamoto et al., 2016). This affective component of body odor declines with age (Okamoto et al., 2016; Croy et al., 2017), which is supported by our results demonstrating that pleasantness drives pre- but not postpubertal classification. The latter was predicted by higher body odor intensity, which has been associated with less positive perception (Doty et al., 1978). In the sense of the mother-child relationship, this leads us to speculate that the intensity drives an avoidant reaction to postpubertal body odors. Hence, this could be interpreted as a mechanism for detachment, when the child becomes more independent and separates itself from parental care (Beyers et al., 2003).

## Conclusion

In summary, this study demonstrates that developmental information is transcribed in body odor across childhood. While prepubertal status is generally transmitted and characterized by pleasant perception, postpubertal status is rather detected by mothers having expertise with children in that stage, and accompanied by higher intensity ratings. Mothers are further able to encode developmental information for classification when assessing body odors with similar developmental status to their own child. As the composition of body odor is still poorly understood, it remains to be determined how chemicals manifest body odor and how they actually influence olfactory perception.

## Data Availability Statement

The datasets generated for this study are available on request to the corresponding author.

## Ethics Statement

The studies involving human participants were reviewed and approved by Ethikkommission TU Dresden. Written informed consent to participate in this study was provided by the participants’ legal guardian/next of kin.

## Author Contributions

IC, AS, and LS contributed to the conception and design of the study. LS acquired the data and wrote the first draft of the manuscript. LS and IC performed the statistical analysis. IC wrote the sections of the manuscript. AS and KW critically revised the manuscript. All authors contributed to the manuscript revision, and read and approved the submitted version.

## Conflict of Interest

The authors declare that the research was conducted in the absence of any commercial or financial relationships that could be construed as a potential conflict of interest.

## Footnotes

1 www.dm.de

2 www.eubos.de

3 https://www.socscistatistics.com/tests/chisquare2/default2.aspx
