# Supplementary material for: Children’s Body Odors: Hints to the Development Status
Source: Front Psychol. 2020 Mar 4;11:320. doi: 10.3389/fpsyg.2020.00320 (PMC7064733; doi:10.3389/fpsyg.2020.00320)
Supplement: Supplementary file 1 [file Data_Sheet_1.pdf]

Supplementary material: Children´s body odors: Hints to the development status

Laura Schäfer[1*], Agnieszka Sorokowska[1,2], Kerstin Weidner[1], Ilona Croy[1]

[1] Department of Psychotherapy and Psychosomatic Medicine, Technische Universität Dresden, Germany

[2] Institute of Psychology, University of Wroclaw, Poland

*** Correspondence:**
Laura Schäfer
laura.schaefer@uniklinikum-dresden.de

1 Supplementary Figures

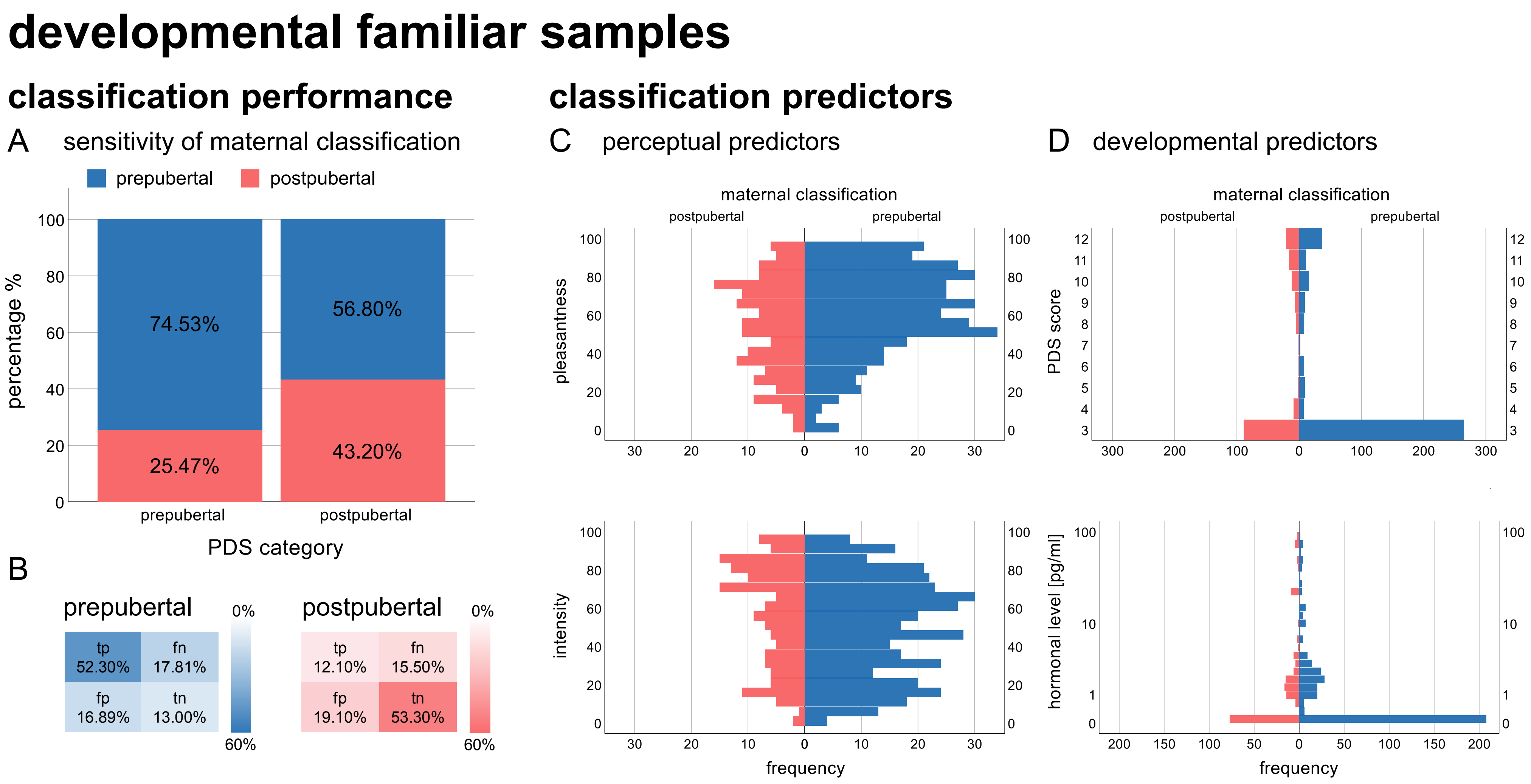

Supplementary Figure 1. Left panel: Classification performance for developmental familiar samples: A: Percentage of the sensitivity of maternal classification plotted by PDS categories; B: Percentage of frequency of true positives (tp), false positives (fp), false negatives (fn) and true negatives (tn) plotted in blue for prepubertal and in read for postpubertal body odors. Color intensity indicates frequency of choice. Right panel: Classification predictors: C: perceptual predictors (above: pleasantness, below: intensity); D: developmental predictors (above: pubertal development scale (PDS), below: hormonal concentration in pg/ml, estradiol for girls, testosterone for boys). Note: Assessment of developmental predictors was carried out for all children from the age of 5 years on and therefore children under the age of 5 exhibit a value of 3 for the PDS (prepubertal) and a value of 0 for the hormonal concentration.

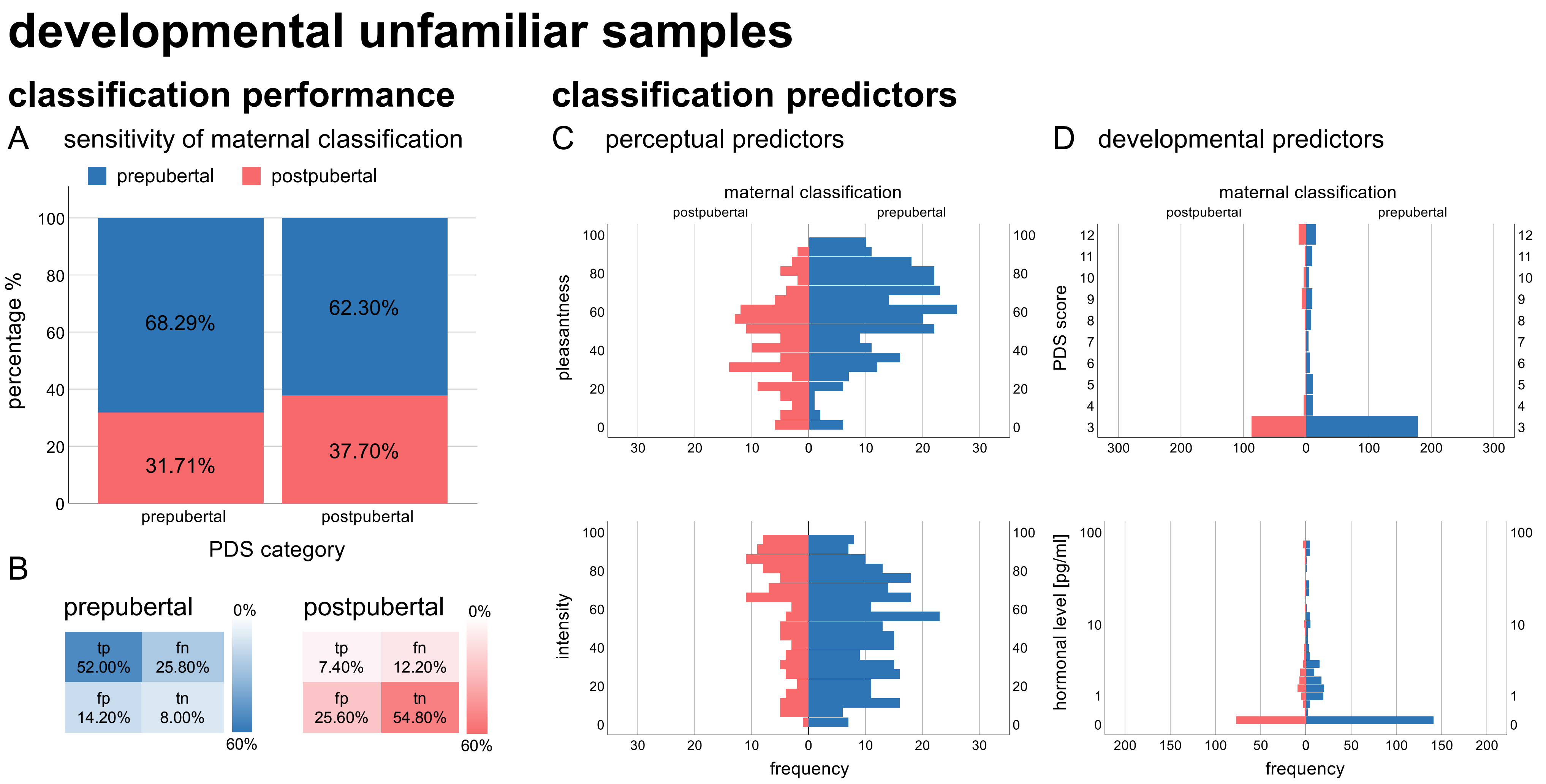

Supplementary Figure 2. Left panel: Classification performance for developmental unfamiliar samples: A: Percentage of the sensitivity of maternal classification plotted by PDS categories; B: Percentage of frequency of true positives (tp), false positives (fp), false negatives (fn) and true negatives (tn) plotted in blue for prepubertal and in read for postpubertal body odors. Color intensity indicates frequency of choice. Right panel: Classification predictors: C: perceptual predictors (above: pleasantness, below: intensity); D: developmental predictors (above: pubertal development scale (PDS), below: hormonal concentration in pg/ml, estradiol for girls, testosterone for boys). Note: Assessment of developmental predictors was carried out for all children from the age of 5 years on and therefore children under the age of 5 exhibit a value of 3 for the PDS (prepubertal) and a value of 0 for the hormonal concentration.

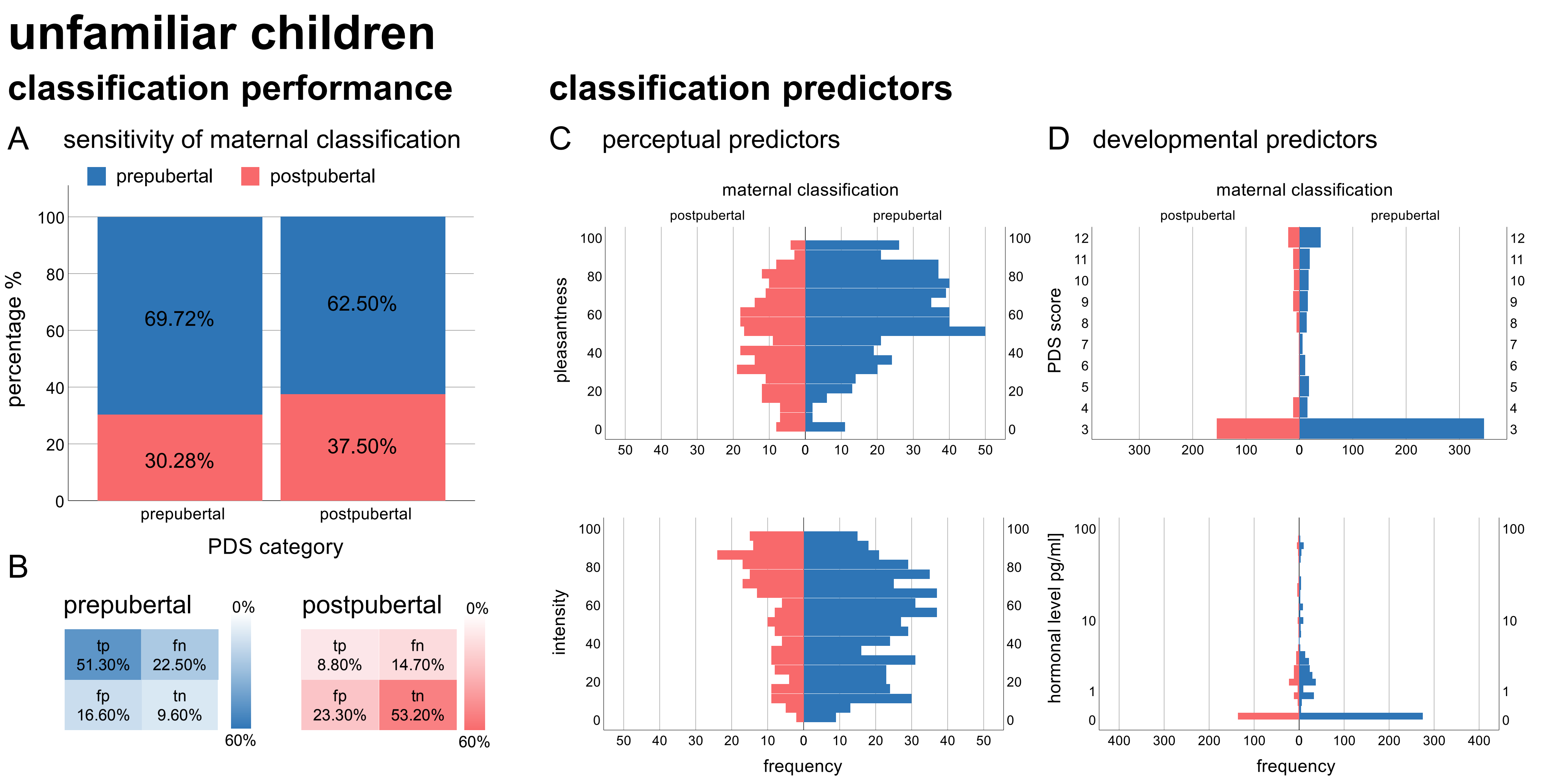

Supplementary Figure 3. Left panel: Classification performance for all unfamiliar children: A: Percentage of the sensitivity of maternal classification plotted by PDS categories; B: Percentage of frequency of true positives (tp), false positives (fp), false negatives (fn) and true negatives (tn) plotted in blue for prepubertal and in read for postpubertal body odors. Color intensity indicates frequency of choice. Right panel: Classification predictors: C: perceptual predictors (above: pleasantness, below: intensity); D: developmental predictors (above: pubertal development scale (PDS), below: hormonal concentration in pg/ml, estradiol for girls, testosterone for boys). Note: Assessment of developmental predictors was carried out for all children from the age of 5 years on and therefore children under the age of 5 exhibit a value of 3 for the PDS (prepubertal) and a value of 0 for the hormonal concentration.

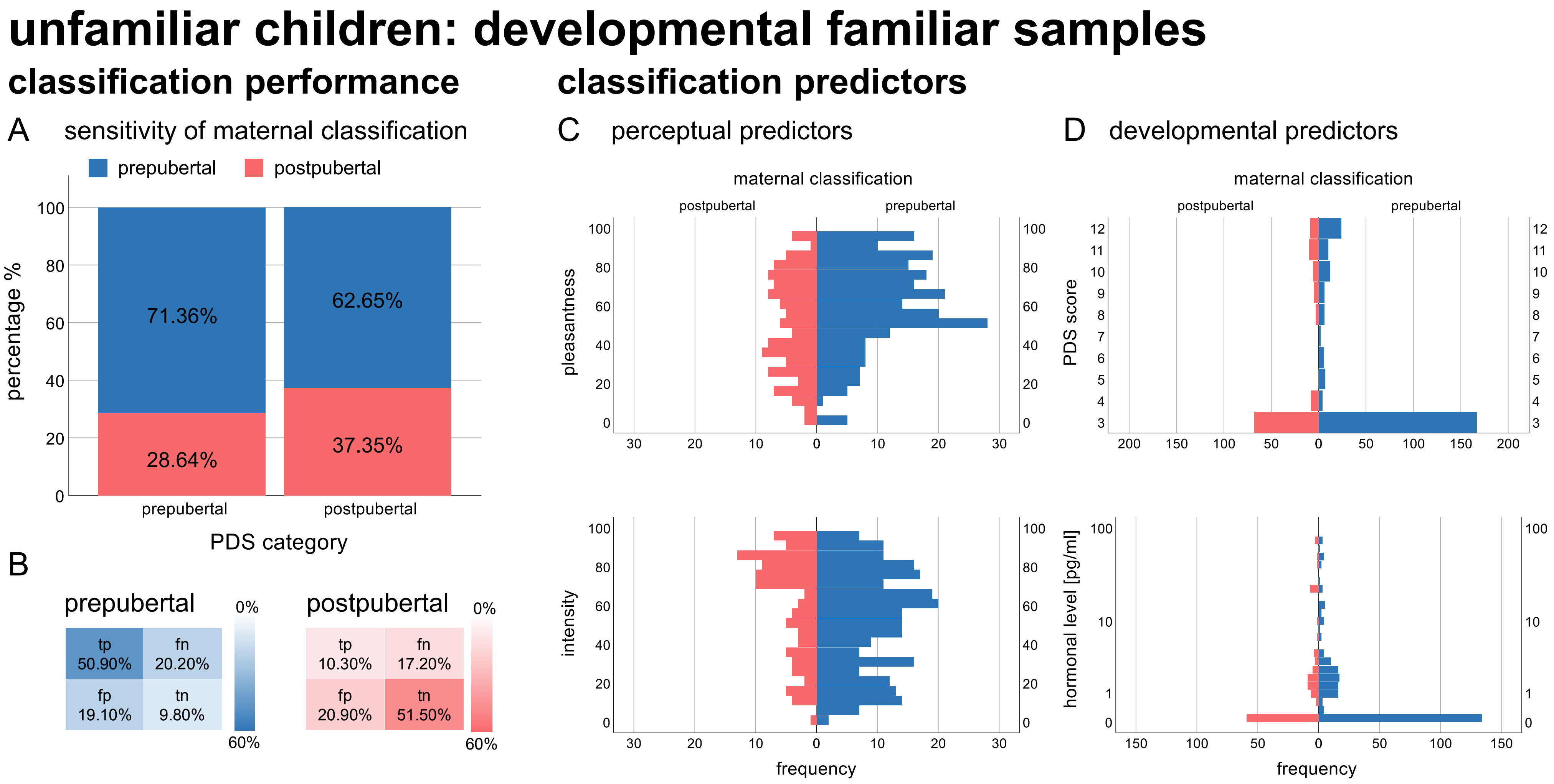

Supplementary Figure 4. Left panel: Classification performance for developmental familiar samples of unfamiliar children: A: Percentage of the sensitivity of maternal classification plotted by PDS categories; B: Percentage of frequency of true positives (tp), false positives (fp), false negatives (fn) and true negatives (tn) plotted in blue for prepubertal and in read for postpubertal body odors. Color intensity indicates frequency of choice. Right panel: Classification predictors: C: perceptual predictors (above: pleasantness, below: intensity); D: developmental predictors (above: pubertal development scale (PDS), below: hormonal concentration in pg/ml, estradiol for girls, testosterone for boys). Note: Assessment of developmental predictors was carried out for all children from the age of 5 years on and therefore children under the age of 5 exhibit a value of 3 for the PDS (prepubertal) and a value of 0 for the hormonal concentration.

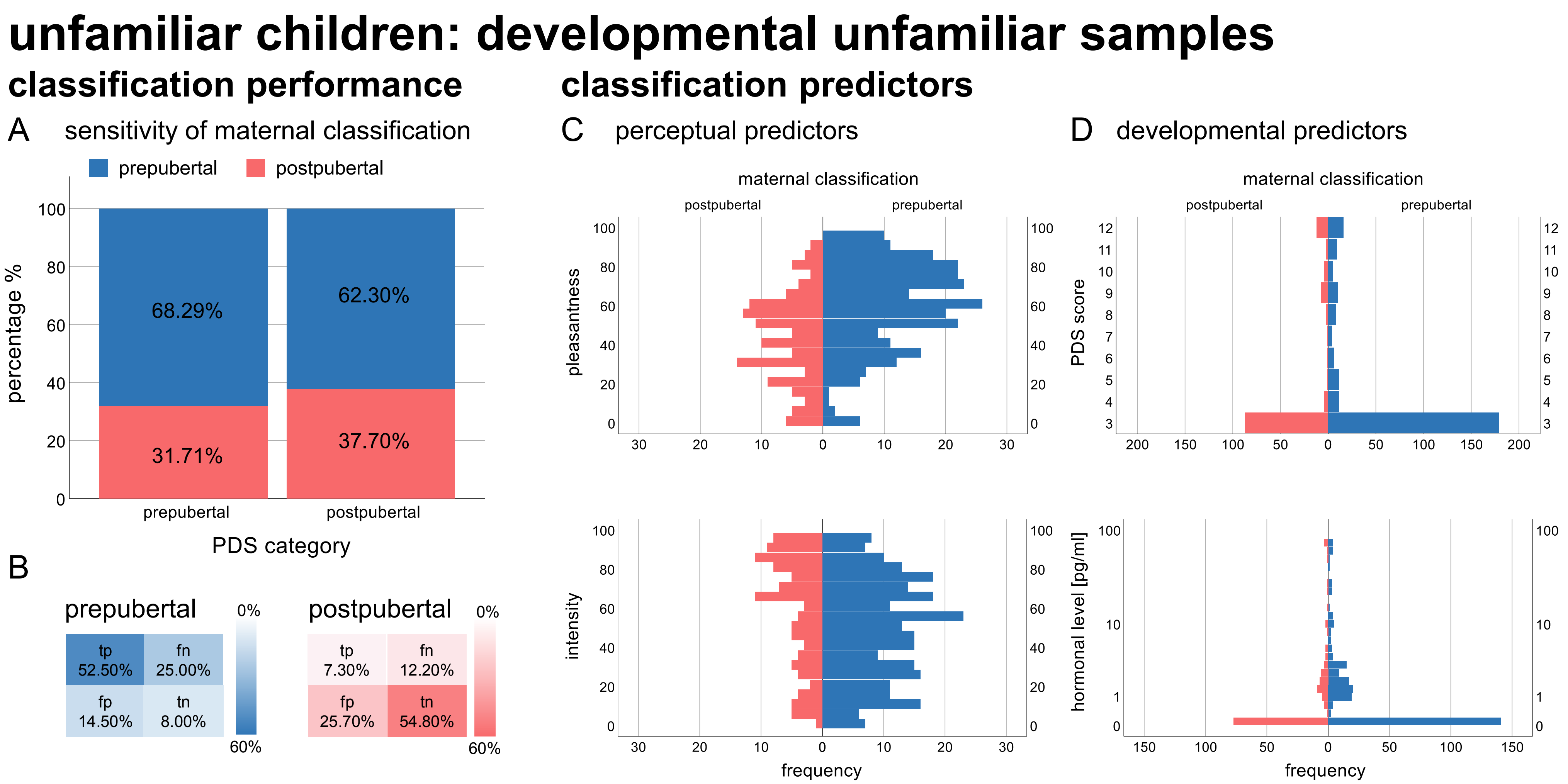

Supplementary Figure 5. Left panel: Classification performance for developmental unfamiliar samples of unfamiliar children: A: Percentage of the sensitivity of maternal classification plotted by PDS categories; B: Percentage of frequency of true positives (tp), false positives (fp), false negatives (fn) and true negatives (tn) plotted in blue for prepubertal and in read for postpubertal body odors. Color intensity indicates frequency of choice. Right panel: Classification predictors: C: perceptual predictors (above: pleasantness, below: intensity); D: developmental predictors (above: pubertal development scale (PDS), below: hormonal concentration in pg/ml, estradiol for girls, testosterone for boys). Note: Assessment of developmental predictors was carried out for all children from the age of 5 years on and therefore children under the age of 5 exhibit a value of 3 for the PDS (prepubertal) and a value of 0 for the hormonal concentration.

2 Supplementary Results

We explored our hypotheses additionally for all unfamiliar children (excluding the own child´s body odor sample from analyses).

2.1 Mothers are able to accurately distinguish pre- from postpubertal odors (H1)

When presented to body odors of prepubertal children, mothers stated in 69.7% of the cases that those odors were from a prepubertal donor. When presented to body odors of postpubertal children, mothers stated in 62.5% of the cases that those odors were from a prepubertal donor and in turn, mothers stated in 37.5% of the cases that the odors were from a postpubertal donor (see Supplementary Figure 3). This result indicates that body odor classification towards postpubertal status tended to increase with developmental stage of the child, although this was not significant ($\chi^2(1) = 2.62$, $p = .105$). Furthermore, this result reveals that body odors are more frequently rated as originating from a prepubertal than from a postpubertal donor.

Mothers classified prepubertal body odors with an accuracy of 60.8%. This value exceeds the 50% chance level. However, the RATZ-index of 0.07 is rather low and suggests that mothers do not perform substantially better than chance. Maternal assessments of prepubertal odors had a sensitivity of 69.5% at a specificity of only 36.7%, indicating that maternal assessments tended to accept the classifying of a sample as prepubertal ($\chi^2(1) = 385.83$, $p < .001$).

A similar effect was found for postpubertal BOs, which were detected with an accuracy of 61.9% at an RATZ-index of 0.08. Maternal assessments of postpubertal odors had a sensitivity of only 37.5% and a specificity of 69.5%, indicating that maternal assessments tended to reject the classification of a sample as postpubertal.

2.2 Classification ability depends on developmental familiarity of the mothers (H2)

Separate analyses of developmental familiar samples and developmental unfamiliar samples revealed that mothers of prepubertal children could identify prepubertal odors with an accuracy of 61.2% (RATZ-index = 0.09; sensitivity = 69.5%; specificity = 35.1%) compared to the 61.8% accuracy of mothers having postpubertal children (RATZ-index: 0.07%; sensitivity = 67.7%; specificity= 35.7%; see Supplementary Figures 3, 4). Classification did not differ significantly between the groups (developmental familiar samples vs. developmental unfamiliar samples: $\chi^2(1) = 5.56$, $p = .135$).

Similarly, both groups did not differ between classification performance of postpubertal body odors (developmental familiar samples: accuracy = 60.2%, RATZ-index: 0.09; sensitivity = 28.4%; specificity = 77.5; developmental unfamiliar samples: accuracy = 60.6%, RATZ-index: 0.07%; sensitivity = 37.7%; specificity = 68.1%; developmental familiar samples vs. developmental unfamiliar samples: $\chi^2(1) = 5.97$, $p = .113$).

2.3 Predictors of pre- vs. postpubertal BO classification (H3)

The overall regression model across all unfamiliar children was significant $\chi^2(4) = 78.40$, $p < .001$. Pleasantness ($p < .001$) and intensity ($p < .001$) significantly predicted age classification with higher pleasantness being associated with prepubertal classification and higher intensity relating to

postpubertal classification (see Supplementary Table 1, Supplementary Figure 3). No other predictor contributing to developmental classification was found.

The overall further regression models testing the respective groups were significant for developmental familiar samples ($\chi^2(4) = 50.1$, p <.001) and for developmental unfamiliar samples ($\chi^2(4) = 50.28$, p <.001). For both groups, perceptual ratings significantly predicted classification: pleasantness (developmental familiar samples: p <.001; developmental unfamiliar samples: p < .001) with higher pleasantness predicting prepubertal classification, as well as intensity (developmental familiar samples: p <.001; developmental unfamiliar samples: p = .001), indicating that higher intensity related to postpubertal identification (see Supplementary Tables 2, 3 and Supplementary Figures 4, 5).

Supplementary Tables

Supplementary Table 1. *Results of logistic regression model predicting age classification; β, SE, Wald, df, p, e^β, 95% CI (e^β) of each predictor: all samples*

**Predictor**	*β*	*SE β*	*Wald's χ^2*	*df*	*p*	e^β	95% CI (e^β)	
Pleasantness	-.022	.004	39.144	1	.000	.978	.972	.985
Intensity	.016	.003	24.628	1	.000	1.016	1.009	1.022
Pds	.012	.028	.197	1	.657	1.013	.958	1.070
Hormones	-.002	.007	.049	1	.825	.998	.985	1.012
Constant	-.539	.321	2.828	1	.093	.583		

Note. R^2 = .10 (Cox & Snell) .14 (Nagelkerke). Model $\chi^2(4) = 78.40$, p <.001.

Supplementary Table 2. *Results of logistic regression model predicting age classification; β, SE, Wald, df, p, e^β, 95% CI (e^β) of each predictor: developmental familiar samples*

**Predictors**	*β*	*SE β*	*Wald's χ^2*	*df*	*p*	*Exp(B)*	95% CI (e^β)	
Pleasantness	-.018	.005	13.550	1	.000	.982	.973	.992
Intensity	.016	.005	12.509	1	.000	1.017	1.007	1.026
Pds	.024	.038	.379	1	.538	1.024	.950	1.104
Hormones	.000	.010	.001	1	.975	1.000	.982	1.019
Constant	-.873	.463	3.554	1	.059	.418		

Note. R^2 = .08 (Cox & Snell) .12 (Nagelkerke). Model $\chi^2(4) = 50.1$, p <.001.

Supplementary Table 3. *Results of logistic regression model predicting age classification; β, SE, Wald, df, p, e^β, 95% CI (e^β) of each predictor: developmental unfamiliar samples*

**Predictors**	*β*	*SE β*	*Wald's χ^2*	*df*	*p*	*Exp(B)*	95% CI (e^β)	
Pleasantness	-.026	.005	26.491	1	.000	.974	.964	.994
Intensity	.014	.004	10.826	1	.001	1.014	1.006	1.023
Pds	.001	.042	.000	1	.990	1.001	.922	1.086
Hormones	-.004	.010	.182	1	.670	.996	.977	1.015
Constant	-.160	.454	.124	1	.725	.852		

Note. R^2 = .12 (Cox & Snell) .14 (Nagelkerke). Model $\chi^2(4) = 50.28$, p <.001
